# Polyphyllin H Reverses Paclitaxel Resistance in Breast Cancer by Binding Membrane Cholesterol to Inhibit Both ABCB1 and ABCC3

**DOI:** 10.3390/ph18111699

**Published:** 2025-11-09

**Authors:** Zheng Ye, Chao Hong, Min Jiang, Wenkui Zou, Yaning Ren, Mingfang Li, Xinyue Xue, Xiaoting Xie, Tong Zhang, Yue Ding

**Affiliations:** 1 School of Pharmacy, Shanghai University of Traditional Chinese Medicine, Shanghai 201203, China; yezheng076@163.com (Z.Y.); jm_caroline@163.com (M.J.); zzzzwk1024@163.com (W.Z.); 13999714660@163.com (M.L.); xuexinyuexxy@163.com (X.X.); xiaoting_0212@126.com (X.X.); 2 State Key Laboratory of Integration and Innovation of Classic Formula and Modern Chinese Medicine, Shanghai University of Traditional Chinese Medicine, Shanghai 201203, China; 3 National Innovation Platform for Medical Industry-Education Integration, Shanghai University of Traditional Chinese Medicine, Shanghai 201203, China

**Keywords:** taxol resistance, transporters, lipid rafts, cholesterol, polyphyllins

## Abstract

**Background/Objectives**: Breast cancer is the most prevalent malignancy among women, and paclitaxel (PTX) is a first-line chemotherapeutic, but chemoresistance driven by ATP-binding cassette (ABC) transporters limits its efficacy. Single-target ABC inhibitors fail due to toxicity and cooperative transporter activity, creating an urgent need for safe multi-target strategies. Membrane cholesterol-rich lipid rafts support ABC transporter function, making cholesterol a key chemoresistance target. This study explored a cholesterol-targeted approach for overcoming PTX resistance. **Methods**: A PTX-resistant breast cancer line (MCF-7/PTX) showing ABCB1/ABCC3 co-upregulation and enriched cholesterol rafts was established. The effects of Polyphyllin H (PPH), a steroidal saponin from *Paris polyphylla*, were compared with lovastatin, a biosynthetic cholesterol inhibitor. In vitro and in vivo assays investigated Polyphyllin H’s cholesterol binding and effects on transporters, PTX accumulation, and tumor growth. **Results**: PPH directly binds membrane cholesterol, disrupting lipid rafts, downregulating ABCB1/ABCC3, reducing drug efflux, and increasing intracellular PTX to restore sensitivity. PPH showed superior cholesterol-binding and resistance-reversal efficacy than lovastatin, with faster, stronger PTX-enhanced cytotoxicity and tumor suppression. **Conclusions**: PPH reverses PTX resistance by targeting cholesterol-lipid rafts to inhibit multiple ABC transporters. This offers a safer adjuvant for PTX-based breast cancer therapy and a translational framework for other drug-resistant malignancies.

## 1. Introduction

Breast cancer remains the predominant malignancy among women worldwide, and its incidence is increasing [1,2]. Paclitaxel (PTX) is a first-line taxane chemotherapeutic agent extensively applied in managing solid malignancies, including breast cancer [3]. However, prolonged or repeated chemotherapy often leads to the development of multidrug resistance (MDR), which significantly compromises therapeutic efficacy and increases the risk of recurrence [4].

MDR is a complex, multifactorial, and multi-pathway phenomenon involving mechanisms, including genetic factors, increased DNA repair capacity, and drug efflux mediated by the ATP-binding cassette (ABC) transporter family, among which the ABC transporter family is the most widely studied [4]. ABC transporters constitute a class of membrane-spanning proteins that utilize ATP hydrolysis to actively export drugs, thereby reducing intracellular drug accumulation [5]. Among them, P-glycoprotein (P-gp, ABCB1) is the most extensively studied and is widely recognized as a key mediator of chemotherapy resistance [6,7]. Although several ABCB1 inhibitors, such as tariquidar [8], zosuquidar [9,10], and elacridar, have been developed in recent years, their efficacy in reversing MDR remains limited, and severe toxic side effects have been observed in clinical trials [11]. Consequently, no ABCB1 inhibitor has yet received formal approval for clinical application [12].

Studies have shown that in PTX-resistant breast cancer cells, multiple ABC family proteins may be highly expressed simultaneously; further, simply inhibiting ABCB1 is insufficient to restore the sensitivity of cancer cells to the drug [13]. Other studies have demonstrated that in vivo, compared to the knockout of either BCRP or ABCB1 alone, the simultaneous knockout of both BCRP and ABCB1 leads to a 43-fold increase in drug accumulation [14]. Furthermore, evidence suggests that broad-spectrum inhibitors of ABC transporters demonstrate significantly enhanced efficacy in cancers co-expressing two or more transporter isoforms [15]. These findings highlight the potential of multi-target inhibition—simultaneously suppressing multiple overexpressed ABC transporters and blocking various efflux pathways—to significantly enhance the effectiveness of chemotherapeutic agents. However, the number of currently identified broad-spectrum ABC transporter inhibitors remains limited. Moreover, combination therapies involving multiple specific inhibitors may entail risks such as complex pharmacokinetics, cumulative toxicity, and off-target effects. Therefore, developing safe and effective inhibitors targeting multiple ABC transporters is crucial for reversing chemotherapy resistance in breast cancer.

Accumulating evidence indicates a strong linkage between ABC transporter activity and cholesterol-enriched lipid rafts within the plasma membrane [16,17,18,19]. Lipid rafts stabilize membrane protein conformation, regulate transmembrane transport, and facilitate signal transduction, with their structural integrity being highly dependent on cholesterol [20,21]. Elevated cholesterol levels not only increase membrane thickness and rigidity, trapping drugs within cellular compartments, but also enhance ABC transporters’ drug-binding and efflux activities through modulation of their membrane localization on lipid rafts [20,22]. Consequently, targeting membrane cholesterol to destabilize lipid rafts and indirectly inhibit multiple ABC transporters has emerged as a promising strategy for reversing MDR.

Many studies have found that natural compounds have great potential in reversing MDR [23,24,25,26]. Among them, steroidal saponins have been widely applied and have garnered considerable scientific interest owing to their diverse pharmacological and biological activities, such as cholesterol-lowering, anti-inflammatory, and immunomodulatory effects, as well as their potential in adjuvant therapy [27,28]. In addition, owing to their amphiphilic structure, many saponins exhibit pronounced surface activity and have been employed to enhance cell membrane permeability and modulate plasma membrane fluidity through forming complexes with membrane cholesterol, thereby facilitating the delivery of chemotherapeutic agents to the cytoplasm or nucleus [29]. Polyphyllins, steroidal saponins derived from *Paris polyphylla*, exhibit potent anti-tumor activity and the ability to reverse MDR [30,31]. Our previous studies found that polyphyllin I can bind with cholesterol in the cell membrane and reduce lipid raft expression, increasing the sensitivity of liver cancer to doxorubicin [32]. Therefore, it is speculated that this effect of polyphyllins may have great potential in reversing MDR. This study successfully established a PTX-resistant breast cancer cell model, discovered the abnormal expression of multiple ABC transporters in it, and verified the necessity of co-inhibiting these ABC transporters for reversing MDR. Subsequently, we validated the potent PTX resistance-reversing effect of polyphyllins in vitro/in vivo, and explored the underlying mechanism from the perspective of cholesterol-lipid raft-multiple ABC transporters. These findings provide a novel mechanistic rationale and potential therapeutic strategy for cholesterol-mediated, multi-target reversal of chemotherapy resistance.

## 2. Results

### 2.1. Establishment of PTX-Resistant MCF-7 Cells

**Figure 1:**
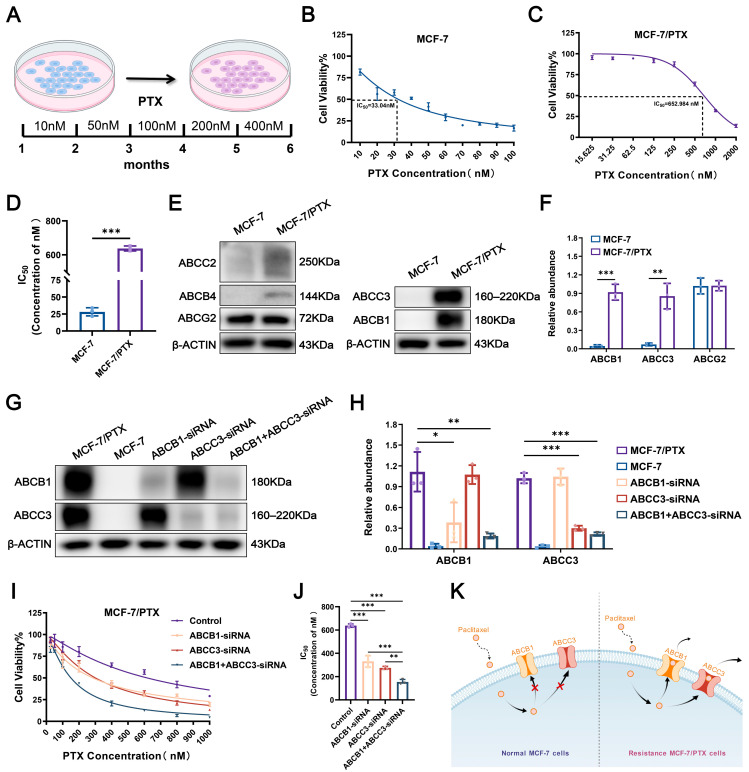
Establishment of the PTX-resistant tumor cell model. (**A**) MCF7/PTX cells were generated by subjecting parental MCF-7 cells to progressively increasing concentrations of PTX over a six-month period. (**B**–**D**) The cytotoxic response to PTX was evaluated in both MCF-7/PTX and MCF-7 cells using the CCK-8 assay, and the corresponding IC_50_ values were determined to assess the degree of acquired MDR. (**E**,**F**) Quantitative analysis of the expression levels of five major ABC transporters was conducted to identify those most significantly altered following the development of resistance. (**G**,**H**) MCF-7/PTX cells were treated with ABCB1-specific siRNA, ABCC3-specific siRNA, or a combination of both, and the protein expression levels of ABCB1 and ABCC3 were assessed via Western blot analysis. (**I**,**J**) MCF-7/PTX cells were treated with ABCB1-specific siRNA, ABCC3-specific siRNA, or a combination of both, and the sensitivity of each group to PTX was evaluated, with the IC_50_ values of PTX determined. (**K**) The key efflux proteins that cause MCF-7/PTX cells to develop drug resistance. Each bar represents the mean ± SD (*n* = 3, * *p* < 0.05, ** *p* < 0.01, *** *p* < 0.001).

Paclitaxel-resistant MCF-7 (MCF-7/PTX) cells were developed by continuously culturing the parental PTX-sensitive MCF-7 cell line in medium supplemented with gradually increasing concentrations of PTX. After six months of stepwise drug induction, a stable drug-resistant subline was successfully established (Figure 1A). These MCF-7/PTX cells exhibited sustained viability and proliferation in the presence of 400 nM PTX. Cytotoxicity was evaluated using the Cell Counting Kit-8 (CCK-8) assay (Figure 1B,C). The half maximal inhibitory concentration (IC_50_) of PTX in the parental MCF-7 cells and MCF-7/PTX cells was 33.04 nM and 652.984 nM, respectively. The IC_50_ value of PTX in MCF-7/PTX cells was approximately 20 times higher than that in the parental MCF-7 cells, thereby confirming the acquisition of a significant PTX-resistant phenotype (Figure 1D).

### 2.2. Activation of Dual-ABC Transporters Leads to PTX Resistance in Tumor Cells

To elucidate the mechanisms underlying PTX resistance, we examined the expression of the five most commonly implicated ABC transporters in both parental MCF-7 and MCF-7/PTX cells (Figure 1E). It is noteworthy that the expression levels of four ABC transporters (ABCB1, ABCC3, ABCB4, and ABCC2) were consistently elevated in MCF-7/PTX cells compared to parental cells. Given that ABCC2 and ABCB4 exhibited much lower expression among these transporters in resistant cells, we prioritized ABCB1 and ABCC3—those showing markedly increased expression—for further investigation, based on their potential role in the development of MDR (Figure 1F). To further evaluate the functional roles of ABCB1 and ABCC3 in PTX resistance, we performed individual and combined knockdown interventions of ABCB1 and ABCC3 in MCF-7/PTX cells, followed by measurement of the IC_50_ of PTX in each group (Figure 1G,H). The analysis revealed that the IC_50_ of PTX in ABCB1-knockdown cells was 281.94 ± 2.450 nM; in ABCC3-knockdown cells, the value was 290.30 ± 2.463 nM. Notably, the combined knockdown of both ABCB1 and ABCC3 resulted in a significant reduction in the IC_50_ to 139.118 ± 2.143 nM, which was much lower than silencing either ABCB1 or ABCC3 alone (Figure 1I,J). This finding suggests that ABCB1 and ABCC3 may cooperatively regulate PTX resistance through a synergistic mechanism, playing a critical role in maintaining the multidrug-resistant phenotype (Figure 1K).

### 2.3. Cholesterol Accumulation Within Lipid Rafts Contributes to the Dysregulation of ABC Transporters

**Figure 2:**
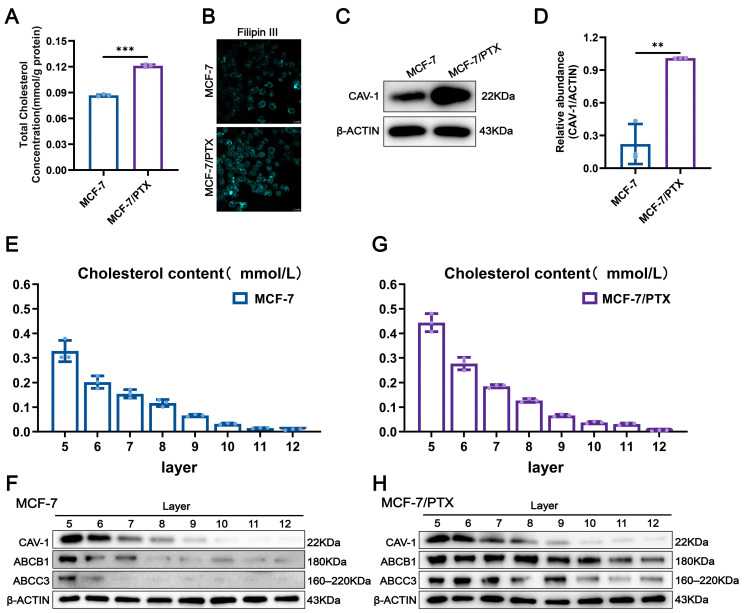
Cholesterol accumulation within lipid rafts contributes to the dysregulation of drug resistance-related membrane proteins. (**A**) Quantification of total cellular cholesterol levels using a Total Cholesterol (TC) Assay Kit to compare differences between MCF-7 cells and MCF-7/PTX cells. (**B**) Assessment of plasma membrane cholesterol levels using Filipin III staining followed by CLSM; fluorescence intensity indicates cholesterol content. (**C**,**D**) Western blot analysis revealed the expression of CAV-1 in MCF-7 cells and MCF-7/PTX cells. (**E**,**G**) Cholesterol content within lipid rafts in MCF-7 cells and MCF-7/PTX cells. (**F**,**H**) Distribution of CAV-1 and ABCB1 across different fractions of MCF-7 cells and MCF-7/PTX cells. Each bar represents the mean ± SD (*n* = 3, ** *p* < 0.01, *** *p* < 0.001).

Previous studies have demonstrated that the total cholesterol content is markedly elevated in drug-resistant cells compared with drug-sensitive counterparts. In the present study, we applied a total cholesterol (TC) assay kit to quantitatively compare cholesterol levels between the MCF-7/PTX cells and MCF-7 cells. The results revealed a marked increase in total cholesterol content in MCF-7/PTX cells (Figure 2A).

To further demonstrate the changes in cholesterol levels between the two types of cells, we employed confocal laser scanning microscope (CLSM) coupled with Filipin III staining. The fluorescence signal indicated that the cholesterol accumulation was predominantly localized to the plasma membrane region (Figure 2B). Consistently, we observed significantly upregulated expression of Caveolin-1 (CAV-1), a classical lipid raft marker protein, in MCF-7/PTX cells compared with MCF-7 cells (Figure 2C,D)

Cholesterol, as a structural component of lipid rafts, is essential for maintaining raft integrity and protein anchoring. Its accumulation leads to increased membrane thickness, which in turn alters membrane fluidity and permeability, thereby modulating the structural and functional integrity of raft domains. We next isolated lipid rafts using sucrose density gradient ultracentrifugation and identified protein-enriched fractions via BCA protein quantification. Cholesterol distribution across the gradient fractions was also determined. Compared with MCF-7 cells, the cholesterol content in each layer of MCF-7/PTX cells after separation was significantly elevated (Figure 2E,G). Interestingly, in MCF-7 cells, ABC transporters were predominantly colocalized with lipid rafts. In MCF-7/PTX cells, raft-associated fractions ranged from layers 5 to 9. ABC transporters were detected in fractions 5–12, they remained most enriched in the cholesterol-dense raft regions, suggesting that their efflux activity may be dependent on raft structural integrity and cholesterol enrichment (Figure 2F,H).

Collectively, these data suggest that acquisition of MDR in MCF-7 cells is accompanied by elevated membrane cholesterol levels, which promote the expansion of lipid raft domains and enhance their function as platforms for ABC transporters. Targeting cholesterol homeostasis in rafts may thus represent a viable strategy to modulate transporter localization and overcome chemoresistance.

### 2.4. PPH Binds with Membrane Cholesterol to Reverse PTX Resistance

**Figure 3:**
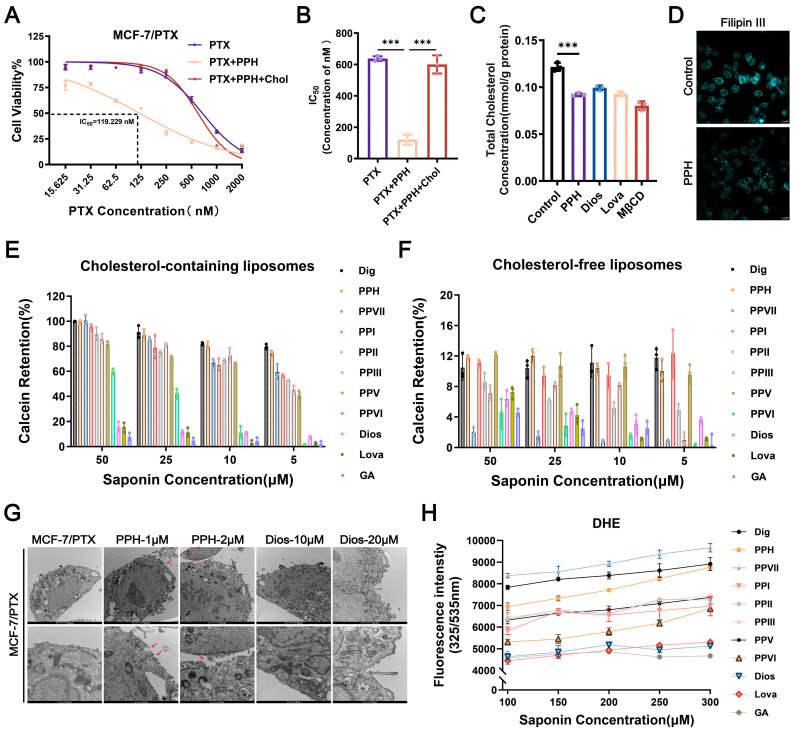
PPH reverses MDR by targeting membrane cholesterol. (**A**,**B**) MCF-7/PTX cells were treated for 48 h with either PTX alone, PTX combined with PPH (1 μM), or PTX combined with PPH (1 μM) and exogenous cholesterol (200 μM). Cell viability was assessed using the CCK-8 assay. (**C**) Cells were treated with lovastatin (5 μM), MβCD (2 mM), or PPH (1 μM) for 48 h, after which intracellular cholesterol levels were quantified using a TC assay kit. (**D**) Changes in plasma membrane cholesterol content after PPH treatment were visualized via Filipin III staining and analyzed using CLSM. (**E**,**F**) Calcein-loaded liposomes containing or lacking cholesterol were incubated with various concentrations of PPH to assess membrane permeability, as indicated by the extent of fluorescence leakage. (**G**) Ultrastructural alterations in the plasma membrane induced by PPH were examined using TEM, with the sites of membrane changes indicated by red arrows. (**H**) The binding interaction between PPH and cholesterol was evaluated using DHE; Dig served as the positive control, while GA functioned as the negative control. Each bar represents the mean ± SD (*n* = 3, *** *p* < 0.001).

In this study, the antitumor activity of eight commercially available polyphyllins was evaluated (Figure S2). Their ability to reverse PTX resistance in MCF-7/PTX cells was further evaluated, with lovastatin used as a positive control (Figure S3, Table S1). The chemical structures of the compounds involved are shown in Figure S1. Among the candidate drugs, PPH combined with PTX had the most significant reversal effect, and the effect was stronger than that of lovastatin combined with PTX (Figure S4). The IC_50_ value of PTX under combination treatment was 119.229 ± 2.076 nM, which was significantly lower than PTX treatment alone, with a resistance reversal fold of 5.5 (Figure 3A,B).

When free cholesterol was added to the combined treatment of PPH and PTX, the resistance-reversing effect of PPH was markedly attenuated in the presence of exogenous cholesterol, indicating a close association between the anti-resistance activity of PPH and cholesterol (Figure 3A,B). Due to the differences in chemical structure between diosgenin (Dios) and PPH, and although Dios has certain reversing MDR activity, its effect is much lower than that of PPH. In order to further explore the mechanism of PPH in reversing MDR by affecting cholesterol, the sugar-free chain structure of Dios was used for compare with PPH to clarify the specific contribution and mechanism of the sugar chain in polyphyllin-mediated reversal of MDR.

Using a TC Assay Kit, we found that both PPH and Dios significantly reduced intracellular cholesterol levels in MCF-7/PTX cells, with efficacy comparable to that of the cholesterol synthesis inhibitor lovastatin (Figure 3C). Considering that approximately 80% of cellular cholesterol is localized in the plasma membrane, we further evaluated membrane cholesterol using Filipin III staining. PPH treatment significantly reduced membrane cholesterol fluorescence in resistant cells, indicating cholesterol extraction (Figure 3D).

To validate the specificity of PPH’s interaction with membrane cholesterol, calcein-loaded liposomes containing cholesterol (or cholesterol-free) were constructed to mimic cell membranes. Both types of liposomes exhibit appropriate particle sizes and surface charge, with uniform dispersion (Table S2). In cholesterol-containing liposomes, PPH induced significant calcein release in a dose-dependent manner, comparable to digitonin (Dig). No significant leakage was observed in cholesterol-free liposomes (Figure 3E,F). Transmission electron microscopy (TEM) demonstrated that PPH caused membrane integrity disruption in a concentration-dependent manner. These morphological changes were not observed in the Dios-treated group (Figure 3G). These findings suggest that PPH exerts its reversal effect by extracting membrane cholesterol, thereby destabilizing membrane structure and enhancing permeability.

We examined the binding capacity of saponins to cholesterol using the fluorescent cholesterol analog dehydroergosterol (DHE). Results indicated that PPH, along with polyphyllins I, II, and VII, exhibited various degrees of DHE-binding, with PPH showing an affinity similar to the positive control Dig. The fluorescence intensity exhibited a concentration-dependent increase, indicating the formation of a stable PPH–cholesterol complex. In contrast, Dios showed no significant DHE fluorescence changes, similar to the negative control glycyrrhizic acid (GA), suggesting a lack of direct cholesterol-binding capacity (Figure 3H). All of these results support the conclusion that PPH binds to cholesterol and extracts it to disrupt membrane integrity.

### 2.5. PPH Targets Cholesterol-Enriched Lipid Rafts to Inhibit Dual-ABC Transporters

**Figure 4:**
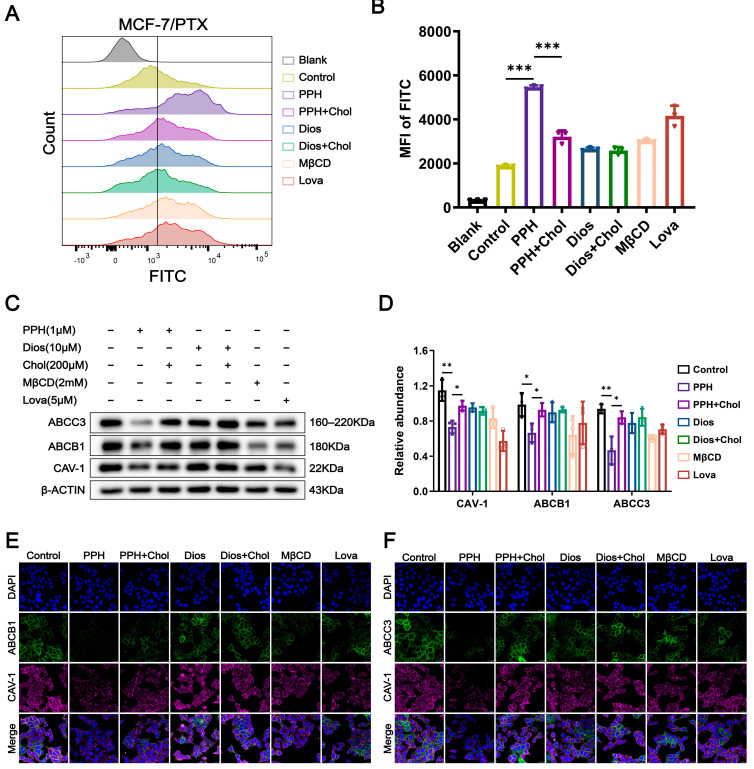
PPH attenuates the overexpression of ABC transporters by modulating lipid raft integrity. (**A**,**B**) After treating MCF-7/PTX cells with FITC-PTX (100 nM) alone or in combination with PPH (1 μM), Dios (10 μM), cholesterol (200 μM), MβCD (2 mM), and lovastatin (5 μM) for 48 h, the accumulation of FITC-PTX within the cells was detected. (**C**,**D**) MCF-7/PTX cells were treated for 48 h with PPH (1 μM), Dios (10 μM), cholesterol (200 μM), MβCD (2 mM), lovastatin (5 μM), or their respective combinations. Protein levels of CAV-1, ABCB1, and ABCC3 were analyzed by Western blot. (**E**,**F**) Cells subjected to the same treatments as in (**C**) were fixed and immunostained with antibodies against ABCB1, ABCC3, and CAV-1, MβCD and lovastatin served as positive controls. Representative fluorescence images and quantification of relative signal intensity are shown. Data are presented as the mean ± SD from three independent experiments (*n* = 3, * *p* < 0.05, ** *p* < 0.01, *** *p* < 0.001).

To further investigate the effects of PPH and Dios on PTX uptake, we first evaluated the cytotoxicity of FITC-labeled paclitaxel (PTX-FITC) in MCF-7/PTX cells to identify low-toxic concentrations (Figure S5). We then analyzed the intracellular accumulation of PTX-FITC using flow cytometry. Experimental results indicate that PPH treatment significantly increased the intracellular fluorescence intensity of PTX-FITC after 24 h, suggesting that PPH effectively enhances the intracellular retention of PTX-FITC and inhibits its efflux. This effect was notably superior to that observed with the positive control drugs MβCD and lovastatin. When PPH was co-treated with cholesterol, the enhancement of drug intracellular accumulation was partially restored, further suggesting a strong affinity between PPH and cholesterol. In contrast, Dios exhibited a weaker effect on the intracellular accumulation of PTX-FITC, and no significant changes were observed after the addition of free cholesterol, indicating that it does not engage in significant interaction with free cholesterol. This suggests that Dios has a more limited role in regulating the intracellular retention of PTX (Figure 4A,B).

Given the previously observed cholesterol-binding property of PPH, we hypothesized that it may perturb membrane architecture by depleting membrane cholesterol, thereby altering membrane fluidity and the structural stability of cholesterol-rich microdomains such as lipid rafts. These alterations could potentially disrupt the localization and expression of ABC transporters, thereby reducing PTX efflux. To validate the aforementioned hypothesis, we used Western blot analysis to evaluate the protein expression levels of multiple ABC transporters and the lipid raft marker CAV-1 in MCF-7/PTX cells treated with PPH and Dios. The results showed that PPH markedly reduced the expression of CAV-1 and the efflux transporters ABCB1 and ABCC3. This downregulation was partially restored when free cholesterol was added and co-treated with PPH. In contrast, Dios exhibited a weaker regulatory effect on the expression of CAV-1 and the efflux transporters ABCB1 and ABCC3, with no significant changes observed after the addition of free cholesterol (Figure 4C,D). Furthermore, CLSM experiments revealed the colocalization of the efflux proteins ABCB1, ABCC3, and the lipid raft protein CAV-1 in lipid raft regions. In the PPH-treated group, the fluorescence intensity of ABCB1 and ABCC3 colocalized with CAV-1 was significantly reduced, consistent with the Western blot results (Figure 4E,F). These results suggest that PPH disrupts lipid raft structure and interferes with the functional expression of key drug transporters, thereby increasing intracellular drug accumulation.

The above results demonstrate that PPH can interact with membrane cholesterol, reduce membrane cholesterol levels, disrupt lipid raft stability, downregulate the expression of multiple ABC transporters, and reduce the efflux of chemotherapy drugs. This mechanism helps to increase the accumulation of intracellular drugs and provides a promising strategy for overcoming PTX resistance in breast cancer.

### 2.6. The In Vivo Evaluation of PPH Reversing PTX Resistance in MCF-7 Cells

**Figure 5:**
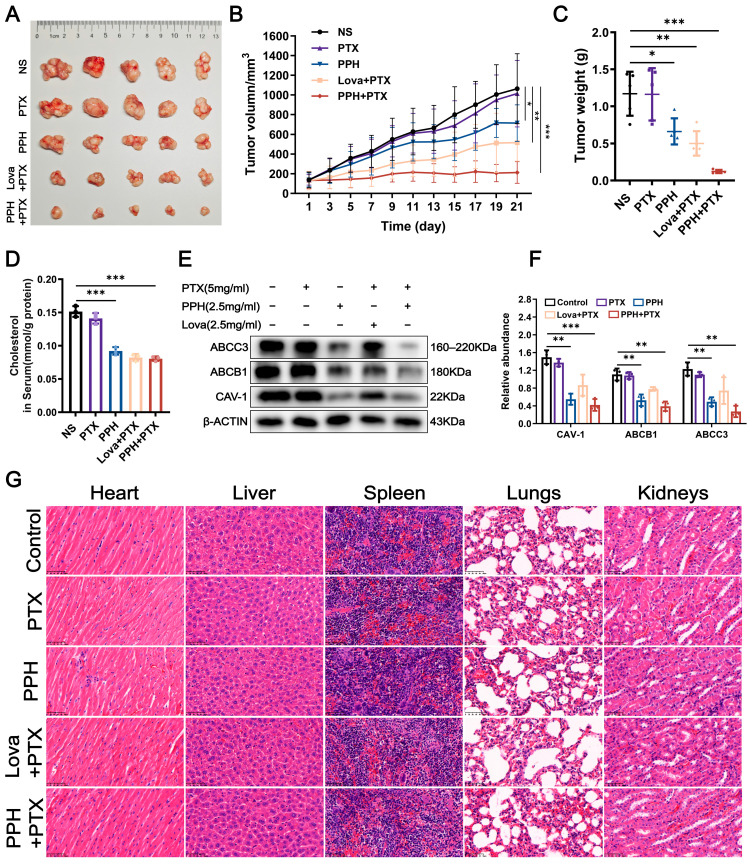
In vivo evaluation of PPH in reversing PTX resistance. (**A**,**B**) MCF-7/PTX cells were subcutaneously inoculated into the right flank region of BALB/c nude mice. When tumor volume reached approximately 100 mm^3^, mice were randomized into five groups and treated with normal saline (NS), PTX (5 mg/kg), PPH (2.5 mg/kg), PTX + PPH (5 mg/kg + 2.5 mg/kg), or PTX + lovastatin (5 mg/kg + 2.5 mg/kg). Tumor growth was monitored every other day during the 21-day treatment period, after which the mice were euthanized. (**C**) Comparison of tumor weights among treatment groups. (**D**) The total cholesterol levels in the tumors of each treatment group. (**E**,**F**) Western blot analysis of ABCB1, ABCC3, and CAV-1 expression in tumor tissues from different groups. Densitometric quantification of band intensities is shown in the accompanying bar graphs. (**G**) H&E-stained tissue sections of the heart, liver, spleen, lungs, and kidneys from each treatment group were examined. All the results are shown as mean ± SD (* *p* < 0.05, ** *p* < 0.01, *** *p* < 0.001).

To evaluate the in vivo antitumor effects of PPH in combination with PTX on PTX-resistant breast cancer, a subcutaneous xenograft model of MCF-7/PTX cells was established in nude mice. The successfully established tumor-bearing mice were randomly divided into groups and treated with PTX alone, PPH alone, lovastatin combined with PTX, or PPH combined with PTX. Treatment was administered continuously for 21 days, and tumor volume changes were monitored regularly during the treatment period (Figure 5B). On day 21, the mice were sacrificed, and the subcutaneous tumors were excised and weighed (Figure 5A,C). The results indicated that the tumor volume growth trend in the PTX monotherapy group was similar to that of the control group, indicating that the model exhibited PTX resistance. In contrast, the PPH combined with PTX treatment group displayed the most significant antitumor effect. The average tumor inhibition rate, calculated based on the final tumor volume of each group, showed that the PPH + PTX group achieved an inhibition rate of approximately 80%, which was significantly superior to the 55% inhibition rate of the lovastatin + PTX group, further supporting the potential advantage of PPH in reversing MDR.

To explore the possible mechanism of action, total cholesterol levels in tumor tissues from different treatment groups were measured. The results indicated that the PPH group, lovastatin + PTX group, and PPH + PTX group all effectively reduced the cholesterol content in the tumor tissues (Figure 5D). Further protein analysis via Western blotting revealed that the expression of CAV-1, ABCB1, and ABCC3 proteins in tumor tissues was significantly downregulated in the PPH + PTX group, with the degree of inhibition stronger than that observed in the lovastatin + PTX group (Figure 5E,F). Additionally, H&E staining results indicated that the tissue structures of the main organs (heart, liver, spleen, lungs, kidneys) in mice from all treatment groups did not show any significant pathological changes, suggesting that the PPH-related treatment regimens had good biosafety at the doses used in this experiment (Figure 5G). In summary, PPH combined with PTX effectively inhibited the in vivo growth of PTX-resistant breast cancer, and its mechanism may be closely related to the downregulation of tumor cholesterol levels and the inhibition of lipid raft-associated transporter protein expression.

## 3. Discussion

PTX resistance continues to pose a major challenge in the treatment of breast cancer and other malignancies. Extensive research has established that drug efflux mediated by ABC transporters constitutes a central mechanism underlying MDR. Among these, the pivotal role of ABCB1 in mediating PTX efflux has been well documented [33,34]. Consistent with these findings, the present study also observed a significant upregulation of ABCB1 in the MCF-7/PTX-resistant cell model. Notably, ABCC3 expression was also significantly elevated in this model, suggesting its potential involvement in PTX resistance. Previous studies have demonstrated that ABCC3 contributes critically to drug resistance across various tumor types. For instance, its overexpression in pancreatic ductal adenocarcinoma (PDAC) cell lines and clinical specimens has identified ABCC3 as a potential therapeutic target in PDAC [35]. Similarly, elevated ABCC3 expression in lung cancer cells has been associated with reduced sensitivity to chemotherapeutic agents such as methotrexate, doxorubicin, vincristine, etoposide, and cisplatin [36,37]. Increasing evidence indicates that tumor resistance often arises from the concurrent overexpression of multiple ABC transporters [38], which may act cooperatively to promote the resistant phenotype. To verify this hypothesis, siRNA-mediated knockdown experiments were performed to individually and simultaneously silence ABCB1 and ABCC3 in MCF-7/PTX cells. The combined silencing of both transporters resulted in the most pronounced restoration of PTX sensitivity, demonstrating that the resistant phenotype of MCF-7/PTX cells is primarily driven by the cooperative function of ABCB1 and ABCC3. Because ABC transporters often share overlapping substrates, inhibition of a single transporter is often insufficient to reverse drug resistance [39,40]. These findings offer mechanistic insight into the limited clinical success of ABCB1 inhibitors, attributing it not only to their intrinsic toxicity and lack of selectivity but also to the compensatory interplay and substrate overlap among multiple ABC transporters. Collectively, our results highlight the importance of considering transporter interplay in the design of therapeutic strategies and underscore the potential of multi-target inhibition approaches for effectively overcoming MDR in cancer therapy [38,41].

Cholesterol is well established as a critical regulator of MDR in tumor cells, influencing essential cellular processes such as membrane composition, signal transduction, and drug efflux [42,43,44]. In this study, we further demonstrated that cholesterol and plasma membrane lipid rafts were significantly enriched in resistant cells, accompanied by elevated expression of CAV-1. Expansion of lipid rafts facilitated the clustering of ABC transporters, which in turn enhanced drug efflux capacity. Previous studies have suggested that membrane cholesterol and raft integrity are fundamental for maintaining the localization and activity of ABC transporters [45,46]. A growing body of research has focused on targeting tumor cholesterol and lipid rafts to combat chemoresistance. Studies have found that depleting raft cholesterol enhances the sensitivity of drug-resistant cells to gefitinib [47]. Simvastatin disrupts the integrity of lipid rafts, inhibits integrin-β3 and focal adhesion formation, thereby enhancing the sensitivity of resistant cells to PTX [48]. Furthermore, depleting membrane cholesterol with MβCD leads to altered localization and loss of function of P-gp [49]. Therefore, targeting cholesterol and lipid raft stability may constitute an effective therapeutic strategy to overcome chemoresistance.

With regard to therapeutic interventions, our findings indicate that the natural steroidal saponin PPH exhibits strong affinity for membrane cholesterol. Unlike lovastatin, which reduces cholesterol levels by inhibiting its biosynthesis, PPH directly binds to and extracts membrane cholesterol, thereby disrupting raft structures and altering the localization and activity of ABCB1 and ABCC3. This mechanism resulted in a significant increase in intracellular PTX accumulation and restored drug sensitivity. The distinction between this “direct membrane-targeting” effect and the “indirect biosynthesis inhibition” accounts for the more rapid and pronounced reversal of resistance observed with PPH in vitro, as well as its stronger tumor-suppressive effect when combined with PTX in vivo. Our findings share notable similarities with those reported for ginsenoside RP1. RP1 has likewise been shown to modulate cholesterol levels and disrupt lipid raft organization, leading to downregulation of P-gp expression and inhibition of Src phosphorylation, thereby reversing the resistance of tumor cells to actinomycin D [50]. Furthermore, our comparative study demonstrated a strong positive correlation between the cholesterol-binding affinity of polyphyllins and their ability to reverse MDR. Among them, PPH exhibited superior cholesterol-binding capacity and PTX resistance reversal compared with Dios, indicating that its unique sugar chain structure likely facilitates enhanced interactions with membrane cholesterol.

Nevertheless, this study is subject to certain limitations. Although we observed upregulation of ABCB1, ABCC3, ABCB4, and ABCC2 in resistant cells, the protein levels of ABCB4 and ABCC2 were comparatively low; therefore, our study primarily focused on the regulation of ABCB1 and ABCC3. Future investigations are required to clarify the potential contributions of ABCB4 and ABCC2 to PTX resistance and to explore their relationship with cholesterol and lipid rafts. Moreover, ABC transporters play an indispensable role in the detoxification of xenobiotics and the function of the blood-brain barrier in normal organs. Excessive or non-targeted depletion of lipid raft cholesterol may lead to a reduction in the basal transport activity of normal cells. Therefore, future strategies that employ tumor-targeted drug delivery systems to restrict the drug’s action to tumor regions will be crucial for ensuring the specificity and safety of the treatment [51,52,53]. In parallel, lipid rafts play a critical role in antigen presentation, as their integrity is essential for MHC-II clustering and the stability of peptide–MHC complexes on antigen-presenting cells (APCs). However, cholesterol depletion disrupts pMHC-II formation and impairs CD4^+^ T-cell activation [54,55,56]. These findings indicate the potential risks of this mode of action to the immune system, which need to be focused on and addressed in our future work.

In conclusion, this study provides evidence that PPH reverses MDR by directly targeting the membrane to impair ABC transporter function and restore drug sensitivity. Unlike conventional broad-spectrum ABC inhibitors, which are limited by toxicity and poor selectivity, targeting cholesterol/lipid raft structures offers a more practical and safer approach to overcoming resistance. If validated in diverse breast cancer subtypes and patient-derived models, this strategy could serve as a promising adjuvant to PTX-based chemotherapy and may be extended to other drug-resistant tumors, thereby offering new translational avenues for the development of clinical MDR-reversal strategies.

## 4. Materials and Methods

### 4.1. Reagents

Paclitaxel, polyphyllin H and other polyphyllin saponins, diosgenin, digitonin and glycyrrhizic acid were purchased from Shanghai Yuanye Biotechnology Co., Ltd. (Shanghai, China). MβCD was obtained from Aladdin Industrial Corporation (Shanghai, China). The CCK-8 assay was purchased from Beyotime Biotechnology Institute (Shanghai, China). The Filipin III staining kit was procured from Med. Chem. Express (Shanghai, China). DHE was purchased from Sigma-Aldrich (Saint Louis, MO, USA). Cholesterol, DOPE and DOPC were purchased from AVT Pharmaceutical Co., Ltd. (Shanghai, China).

### 4.2. Cell Culture

The human breast cancer cell line MCF-7 was purchased from the National Collection of Authenticated Cell Cultures (Shanghai, China). The cells were cultured in DMEM medium supplemented with 10% fetal bovine serum and 1% penicillin-streptomycin (Gibco, Waltham, MA, USA) at 37 °C in a humidified atmosphere containing 5% CO_2_. To establish PTX-resistant cell lines, MCF-7 cells were exposed to increasing concentrations of PTX, starting at 10 nM. Drug-containing medium was added 24 h after cell seeding, and cells were passaged upon reaching 90% confluence. After the passaging of the cells, the concentration was increased by 20 nM. The PTX concentration was continuously increased until the cells exhibited stable proliferation under exposure to 400 nM PTX. This induction process required at least 6 months. The PTX-resistant cell lines were named MCF-7/PTX.

### 4.3. Cell Cytotoxicity Assay

Cell viability was evaluated using the CCK-8 assay. Cells were seeded in 96-well plates at a density of 7 × 10^3^ cells per well and cultured in medium supplemented with varying concentrations of the tested compounds. After 48 h of incubation, 10 μL of CCK-8 solution was added to each well, followed by an additional 1-h incubation. Absorbance was measured at 450 nm using a microplate reader, and the IC_50_ values of each compound were calculated with SPSS 26.0 software.

### 4.4. Western Blot

Total protein was extracted from cultured cells or tumor tissues, and equal amounts of protein were separated by 10% SDS-PAGE. After electrophoresis, proteins were transferred onto polyvinylidene difluoride (PVDF) membranes, which were then blocked with QuickBlock™ Blocking Buffer (Beyotime Biotechnology, Shanghai, China). The membranes were then incubated overnight at 4 °C with the following primary antibodies: anti-ABCB1 (13342, Cell Signaling Technology, Boston, MA, USA), anti-ABCC3 (39909, Cell Signaling Technology, Boston, MA, USA), anti-CAV-1 (66067-1, Proteintech, Wuhan, China), anti-ABCC2 (A8405, ABclonal, Wuhan, China), anti-ABCG2 (A17908, ABclonal, Wuhan, China), anti-ABCB4 (A9835, ABclonal, Wuhan, China), and anti-β-ACTIN (AC026, ABclonal, Wuhan, China). After washing, membranes were incubated for 2 h at room temperature with horseradish peroxidase-conjugated secondary antibodies, including goat anti-mouse IgG (7076, Cell Signaling Technology, Boston, MA, USA) and goat anti-rabbit IgG (7071, Cell Signaling Technology, Boston, MA, USA). Protein bands were visualized using an enhanced chemiluminescence (WBKLS0500, ECL, Millipore, Boston, MA, USA) detection system, and images were captured and quantitatively analyzed with the Luminescent Image Analyzer (AI 600, GE Healthcare Bio-Sciences, Uppsala, Sweden).

### 4.5. Silencing of ABCB1 and ABCC3 Expression by siRNA and Its Effects on Cell Growth and Viability

Specific siRNAs targeting ABCB1 and ABCC3 were used to suppress their expression. According to the manufacturer’s instructions, Opti-MEM^®^ reduced-serum medium was used to prepare the transfection mixtures, which included ABCB1-specific siRNA (HY-RS00048, Med. Chem. Express, Shanghai, China), ABCC3-specific siRNA (HY-RS00062, Med. Chem. Express, Shanghai, China), and the transfection reagent Lipo 3K (APExBIO, Houston, TX, USA). For gene silencing, 2 × 10^5^ cells were plated in 6-well plates containing sterile culture medium. After 24 h of incubation to allow cell adherence, the medium of drug-resistant cells was replaced with fresh medium containing the siRNA transfection mixture, in which ABCB1- and ABCC3-specific siRNAs were diluted in Opti-MEM^®^ to a final siRNA concentration of 5 nM in the culture medium. After 72 h of siRNA incubation, cells were harvested, washed, and reseeded into 96-well plates at a density of 7 × 10^3^ cells per well. After 24 h, the culture medium was replaced with fresh medium containing different concentrations of PTX. After 48 h, cell viability was assessed using the CCK-8 assay to determine the IC_50_, and the sustained knockdown efficiency of ABCB1 and ABCC3 throughout the experiment was verified by Western blot analysis. The detailed transfection sequences of the siRNAs are provided in Table S3.

### 4.6. Immunofluorescent Staining

Cells were seeded onto glass-bottom culture dishes and subjected to the indicated drug treatments. Following three washes with PBS, the cells were fixed with 4% paraformaldehyde for 20 min at room temperature. Non-specific binding sites were blocked by incubating the cells with 2% BSA in PBS for 30 min. Subsequently, the cells were incubated overnight at 4 °C with primary antibodies against ABCB1 (1:500), ABCC3 (1:500), and CAV-1 (1:500) (all from Cell Signaling Technology, Boston, MA, USA). After additional PBS washes, the samples were incubated for 1 h at room temperature with Alexa Fluor 488–conjugated goat anti-rabbit IgG (H + L) and Alexa Fluor 647–conjugated goat anti-mouse IgG (H + L) secondary antibodies (Beyotime Biotechnology, Shanghai, China). Nuclei were counterstained with DAPI for 5 min. Confocal fluorescence images were acquired using a Leica TCS SP8 CLSM.

### 4.7. Extraction and Isolation of Lipid Rafts

MCF-7 and MCF-7/PTX cells (5 × 10^7^ cells each) were harvested and washed three times with ice-cold PBS. The cell pellets were then resuspended in 5 mL of NP-40 lysis buffer and incubated on ice for 30 min. Lysates were centrifuged at 12,000 rpm for 10 min at 4 °C, and the resulting supernatants were collected. Subsequently, 5 mL of 80% sucrose solution was added to each supernatant, mixed thoroughly, and transferred to a 25 mL ultracentrifuge tube. To establish a sucrose density gradient, 10 mL of 35% sucrose solution and 5 mL of 5% sucrose solution were sequentially layered on top. Samples were subjected to ultracentrifugation at 200,000× *g* for 20 h at 4 °C. Following centrifugation, twelve fractions (2 mL each) were carefully collected from top to bottom, and protein samples were prepared from each fraction. The localization of lipid rafts and the expression of ABC transporters were assessed by Western blotting, and the cholesterol level in each fraction was quantified using a cholesterol assay kit.

### 4.8. Filipin III Assays Detect Cell-Membrane Cholesterol Content

Filipin III is a fluorescent probe that specifically binds to unesterified cholesterol and is widely used for the detection of cholesterol in cell membranes. Its maximum excitation wavelengths are 338 nm and 357 nm, with a maximum emission wavelength of 480 nm. In this study, the cholesterol levels in the tumor cell membrane were quantitatively assessed using the Filipin III staining method. Cells were seeded onto glass-bottom culture dishes and maintained under standard culture conditions. After three washes with PBS, the cells were fixed with 4% paraformaldehyde for 20 min at room temperature. The fixed cells were subsequently incubated with Filipin III solution for 30 min at room temperature in the dark. Finally, the fluorescence intensity of the Filipin III staining was measured under excitation at 405 nm using a CLSM, and the changes in cholesterol levels on the tumor cell membrane were analyzed.

### 4.9. Liposome Preparation and Calcein Encapsulation

In this study, two types of liposomes were prepared using the thin-film dispersion method: (1) cholesterol-containing liposomes, with a mass ratio of DOPE: DOPC: cholesterol of 1:6:3; and (2) cholesterol-free liposomes, with a mass ratio of DOPE:DOPC of 1:9. The lipids were dissolved in 4 mL of chloroform and transferred into a round-bottom flask. The solvent was removed by rotary evaporation under reduced pressure at 60 °C for 15 min, yielding a uniform lipid film coating the interior surface of the flask. To eliminate any residual solvents, the film was subsequently subjected to overnight vacuum drying. A 100 mM stock solution of calcein was prepared by dissolving the dye in 1 M NaOH, followed by adjustment of the pH to 7.4. This calcein solution was combined with the dried lipid film, and hydration was performed at 60 °C for 30 min to produce a suspension of liposomes. The liposome dispersion was then sonicated with a SCIENTZ-IID ultrasonic processor (Ningbo Scientz Biotechnology Co., Ltd., Ningbo, China) to decrease particle size and disrupt aggregates. Non-encapsulated calcein was separated from the liposome fraction by size-exclusion chromatography on a Sephadex G-50 gel filtration column (Φ10 mm × 200 mm) that had been thoroughly pre-equilibrated with deionized water. The particle size and zeta potential of the liposomes were further measured to characterize their physical properties. The calcein-loaded liposomes were stored at temperatures below 4 °C, protected from light, until use.

### 4.10. Morphology of Cell Membrane in Transmission Electron Microscope

Collect cells treated with different concentrations of PPH and Dios. For primary fixation, the cells were immersed in 1.5% glutaraldehyde prepared in 0.1 M sodium cacodylate buffer for 2 h at 4 °C. After fixation, they were rinsed three times with the same buffer and then post-fixed in 1% osmium tetroxide prepared in 0.1 M sodium cacodylate buffer for 1 h at 4 °C. Two additional buffer washes were subsequently performed. Dehydration was achieved through a graded ethanol series ranging from 30% to 100%, followed by treatment with absolute propylene oxide. The specimens were then infiltrated with and embedded in Agar-100 resin, which was polymerized by incubation at 60 °C overnight. Ultrathin sections were obtained using an ultramicrotome and examined with a JEOL JEM-1230 transmission electron microscope.

### 4.11. Binding Assay of Polyphyllins with DHE

DHE is a naturally occurring fluorescent analog that closely mimics the physicochemical properties of cholesterol. In this study, DHE was employed as a surrogate for cholesterol in the saponin-binding assay. Saponin solutions at varying concentrations were added to a 1 μg/mL DHE solution, mixed thoroughly, and dispensed into the wells of a fluorescence microplate. Fluorescence intensity was recorded using a microplate reader, and changes in fluorescence signal were used to assess the binding affinity of saponins for cholesterol.

### 4.12. In Vivo Antitumor Effect

To evaluate the in vivo antitumor efficacy of PPH in reversing PTX resistance, female BALB/c nude mice (4 weeks old) were subcutaneously inoculated with 5 × 10^6^ MCF-7/PTX cells in the right axillary region. Once tumor volumes reached approximately 50–100 mm^3^, mice were randomly assigned into five groups (*n* = 5 per group) based on tumor size: (1) control group, (2) PTX (5 mg/kg), (3) PPH (2.5 mg/kg), (4) PTX + PPH (5 mg/kg + 2.5 mg/kg), and (5) PTX + lovastatin (5 mg/kg + 2.5 mg/kg). Drug administration was conducted as follows: PTX was administered via tail vein injection, PPH was delivered intraperitoneally, and lovastatin was given via oral gavage, with a 2-day interval, for a total of 21 days. Tumor volume and body weight were monitored every two days throughout the treatment period. Tumor volume was calculated using the formula: Volume (mm^3^) = (Length × Width^2^)/2. Following the treatment cycle, all mice were euthanized, and the tumors were harvested and weighed.

All experimental animals were purchased from Shanghai Sippr-Bk Lab Animal Co., Ltd. (Shanghai, China) and housed under controlled environmental conditions (temperature 21 ± 1 °C, relative humidity 55 ± 10%) with a 12 h light/dark cycle and unrestricted access to food and water. All animal experiments were conducted in accordance with the guidelines of the Institutional Animal Experimental Ethics Committee of Shanghai University of Traditional Chinese Medicine (SHUTCM) and under the approved protocol (license No. PZSHUTCM2504230003).

### 4.13. Statistical Analysis

All statistical analyses were performed using GraphPad Prism 9.0. Group comparisons were conducted with *t*-test or one-way ANOVA, as appropriate. Data are presented as the mean ± standard deviation (SD). Statistical significance was defined as *p* < 0.05, with specific symbols explained in the figure legends.

## Data Availability

The original contributions presented in the study are included in the article and Supplementary Materials, further inquiries can be directed to the corresponding authors.

## Supplementary Materials

The following supporting information can be downloaded at: https://www.mdpi.com/article/10.3390/ph18111699/s1, Figure S1: The chemical structures of the compounds; Figure S2: 48-h cytotoxicity of Paris saponins and controls in MCF-7/PTX cells; Figure S3: 48-h cytotoxicity of MCF-7/PTX cells treated with PTX (various concentrations) in combination with seven Paris saponins or lovastatin; Figure S4: IC_50_ values of PTX in all combination groups; Figure S5: 48-h cytotoxicity of PTX-FITC in MCF-7/PTX cells; Table S1: Fold reversal of MDR in all combination groups; Table S2: Size and zeta potential of liposomes; Table S3: Sequences of the siRNA.

## Abbreviations

The following abbreviations are used in this manuscript:

CCK-8	Cell counting kit-8
ABC	ATP-binding cassette
MDR	Multidrug resistance
IC_50_	Half maximal inhibitory concentration
P-gp	P-glycoprotein
TC	Total cholesterol
CLSM	Confocal laser scanning microscope
DOPE	1,2-Dioleoyl-sn-glycero-3-phosphoethanolamine
DOPC	1,2-dioleoyl-sn-glycero-3-phosphocholine
PPH	Polyphyllin H
PTX	Paclitaxel
Dios	Diosgenin
Dig	Digitonin
GA	Glycyrrhizic acid
MβCD	Methyl-β-cyclodextrin
Lova	Lovastatin
Chol	Cholesterol
