# Supplementary material for: Polyphyllin H Reverses Paclitaxel Resistance in Breast Cancer by Binding Membrane Cholesterol to Inhibit Both ABCB1 and ABCC3
Source: Pharmaceuticals (Basel). 2025 Nov 9;18(11):1699. doi: 10.3390/ph18111699 (PMC12655514; doi:10.3390/ph18111699)
Supplement: Supplementary file 1 [file pharmaceuticals-18-01699-s001.zip › pharmaceuticals-3925762-supplementary.pdf]

Supplementary Material

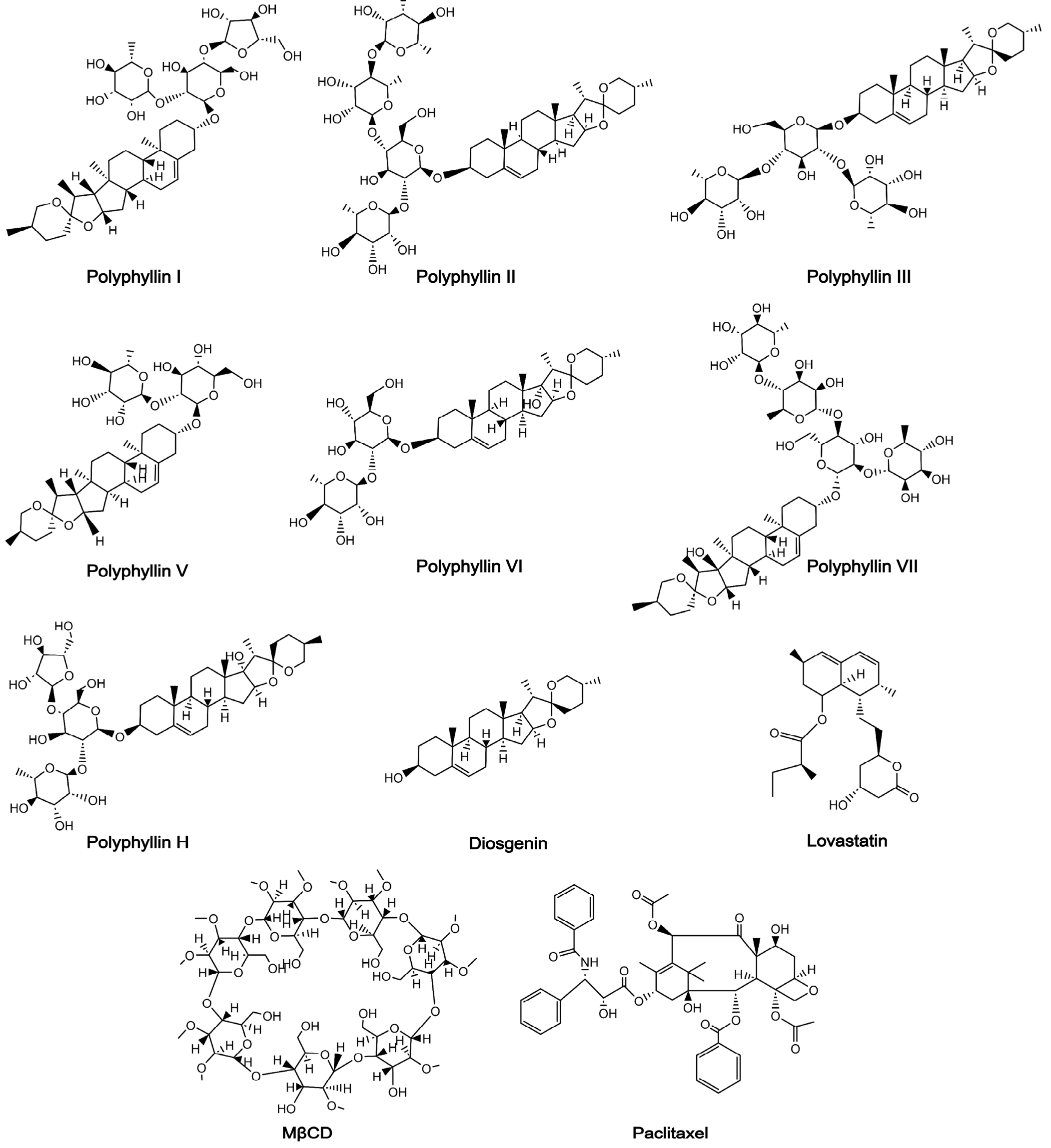

Figure S1. The chemical structures of the compounds.

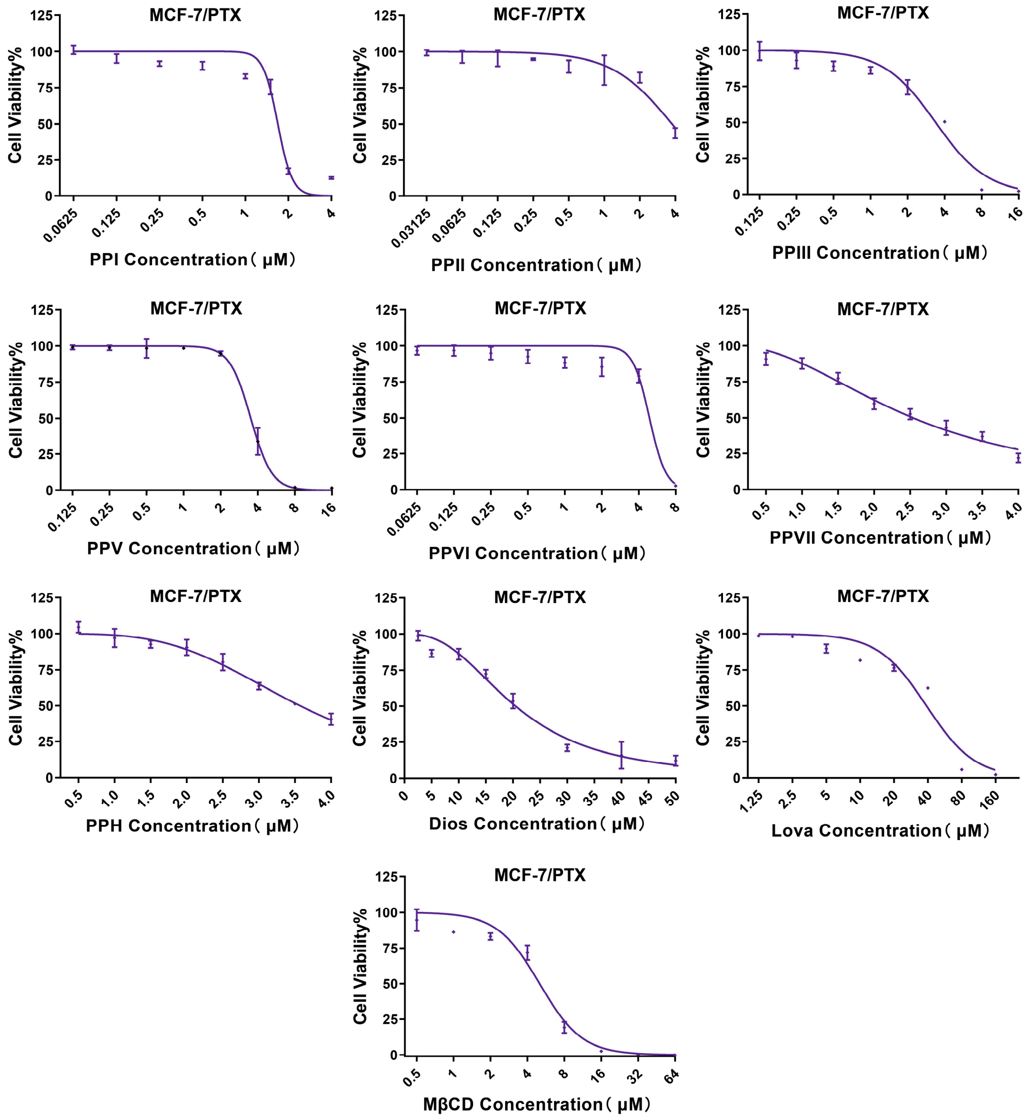

Figure S2. 48-h cytotoxicity of eight polyphyllins, lovastatin, and MβCD in MCF-7/PTX cells measured by the CCK-8 assay to determine safe concentrations with low or no toxicity.

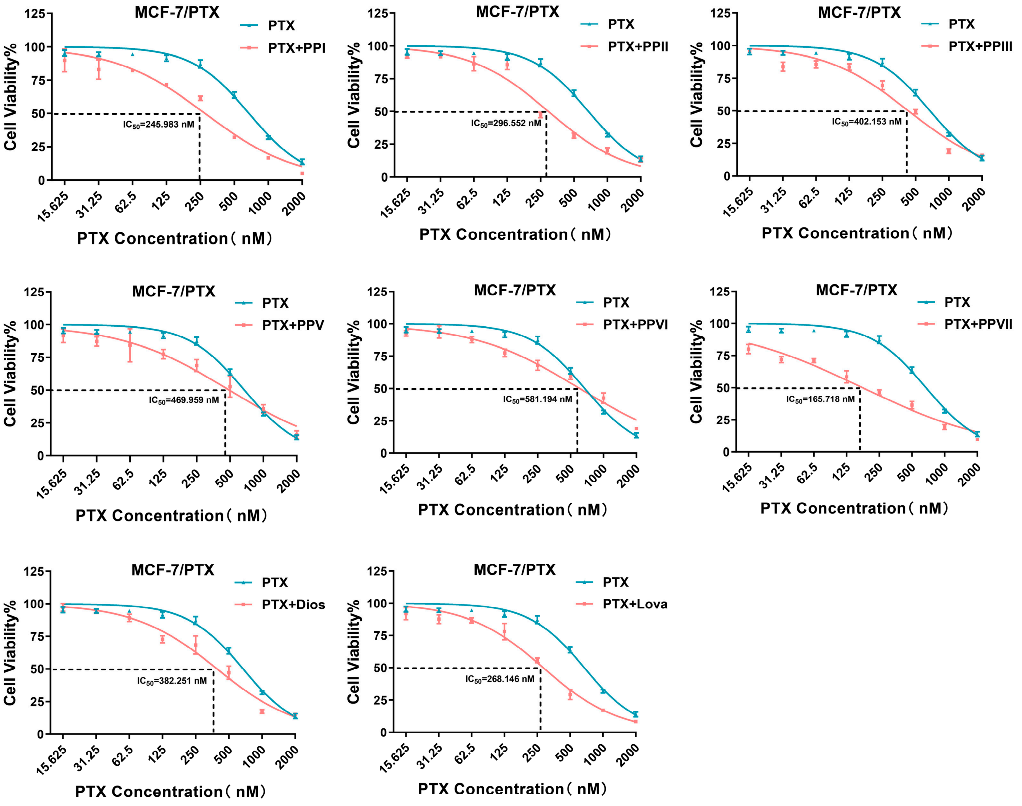

Figure S3. 48-h cytotoxicity of MCF-7/PTX cells exposed to paclitaxel (various concentrations) plus seven Paris saponins, or lovastatin (all at safe concentrations), measured by CCK-8.

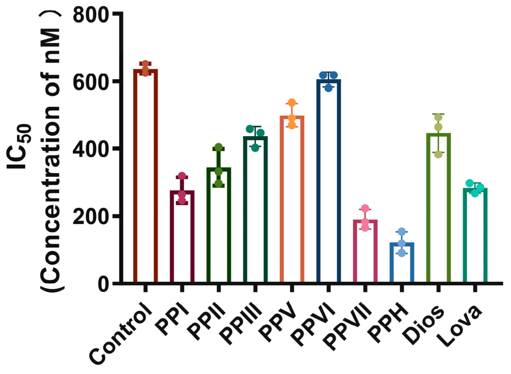

Figure S4. The IC_{50} values of all combinations were calculated with SPSS software. Data are presented as mean ± standard deviation (N = 3).

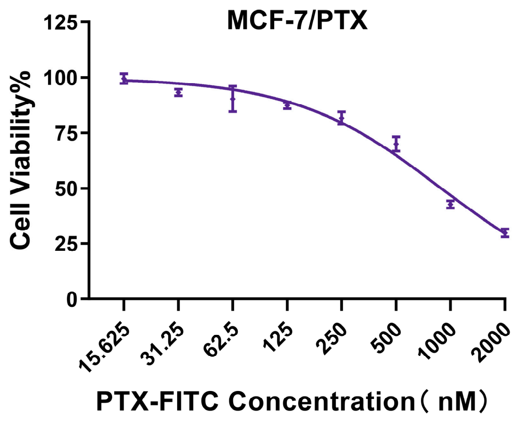

Figure S5. 48-h cytotoxicity of PTX-FITC in MCF-7/PTX cells measured by the CCK-8 assay to determine safe concentrations with low or no toxicity.

Table S1. The IC_{50} values and fold reversal of drug resistance in MCF-7/PTX cells under different combination treatments.

saponins	Safe concentration	IC_{50}（nM）	Reversal of drug resistance fold
PPH	1 μM	119.229 ± 2.076	5.48
PPVII	1 μM	165.718 ± 2.219	3.94
PPI	0.5 μM	245.983 ± 2.391	2.65
Lovastatin	5 μM	268.146 ± 2.428	2.44
PPII	1 μM	296.552 ± 2.472	2.2
Diosgenin	10 μM	382.251 ± 2.582	1.71
PPIII	1 μM	402.153 ± 2.604	1.62
PPV	2 μM	491.722 ± 2.692	1.33
PPVI	2 μM	581.194 ± 2.764	1.23

Table S2. Size and zeta potential of liposomes (mean ± SD).

Liposomes	Size (nm)	PDI	Zeta (mV)
Cholesterol-free liposomes	149.23 ± 1.75	0.24 ± 0.01	3.70 ± 0.66
Cholesterol-containing liposomes	94.55 ± 1.39	0.22 ± 0.01	5.55 ± 0.33

Table S3. Sequences of the siRNA.

Name	Source	Forward (5′ - 3′)	Reverse (5′ - 3′)
ABCB1-siRNA-1	human	AGAAAGAACUUGAAAGGUAUU	UACCUUUCAAGUUCUUUCUUU
ABCB1-siRNA-2	human	GGACAAGCACUGAAAGAUAUU	UAUCUUUCAGUGCUUGUCCUU
ABCB1-siRNA-3	human	UGAAGGAAUUGGUGACAAAUU	UUUGUCACCAAUUCCUUCAUU
ABCC3-siRNA-1	human	AAAGAUGUAGUGGUAAUAGUU	CUAUUACCACUACAUCUUUUU
ABCC3-siRNA-2	human	UGAUGUAGCCACGACAAUGUU	CAUUGUCGUGGCUACAUCAUU
ABCC3-siRNA-3	human	AAGUCUUAAGAUAUUAAACUU	GUUUAAUAUCUUAAGACUUUU
